# Fostering inclusion in clinical and research procedures in autistic youth through augmentative and alternative communication: a narrative review and a proposal for visual aids application in brain stimulation

**DOI:** 10.3389/fpsyt.2026.1764016

**Published:** 2026-07-09

**Authors:** Sara Passarini, Fabio Quarin, Giulia Lazzaro, Floriana Costanzo, Andrea Battisti, Giovanni Valeri, Silvia Guerrera, Laura Casula, Deny Menghini, Sabine Pirchio, Stefano Vicari, Elisa Fucà

**Affiliations:** 1 Child and Adolescent Neuropsychiatry Unit, Bambino Gesù Children’s Hospital, IRCCS, Rome, Italy; 2 Department of Dynamic and Clinical Psychology and Health Studies, Sapienza University of Rome, Rome, Italy; 3 Life Sciences and Public Health Department, Catholic University, Rome, Italy

**Keywords:** autism spectrum disorder, communication boards, equity, ethics, language, tDCS

## Abstract

Autistic children and adolescents often experience medical and neurocognitive co-occurrences that necessitate regular healthcare monitoring. Language difficulties, often observed in autistic individuals, can impede effective communication and participation in medical settings, potentially reducing treatment compliance. Communication barriers also pose challenges in involving autistic youth in research protocols, such as those including Non-Invasive Brain Stimulation (NIBS). Augmentative and Alternative Communication (AAC) strategies could facilitate the participation of autistic individuals in both healthcare and research settings.

This narrative review synthesizes the use of AAC to enhance compliance in healthcare and clinical research contexts for autistic children and adolescents. Findings suggest that AAC aids can reduce stress and anxiety, thereby improving adherence to healthcare protocols. However, no studies were identified that specifically address AAC’s role in enhancing compliance during clinical research procedures involving autistic children and adolescents. Based on the current literature, here we propose a set of AAC aids to improve understanding and compliance with NIBS in autistic individuals.

## 1 Introduction

Autism Spectrum Disorder (ASD) is a neurodevelopmental disorder characterized by social interactions difficulties along with repetitive motor movements, insistence on sameness, focused interests, and/or sensory sensitivities (1). Medical co-occurrences such as gastrointestinal issues, sleep disturbances, metabolic disorders, epilepsy, and immune system anomalies are frequently observed (2–5). Co-occurring conditions required regular healthcare checkups (2), which should be conducted using a multidisciplinary and integrated approach (2).

Nonetheless, autistic individuals often face significant barriers, such as physical and social barriers, which hinder their access to timely and appropriate healthcare (6). Healthcare access barriers are experienced across the lifespan, from childhood through adulthood (7). Indeed, families of autistic children frequently report substantial difficulties in obtaining needed care. For example, autistic youth aged 10–17 years seem to be less likely to receive preventive services, such as annual influenza vaccination (8). Further, autistic adults described difficulties in accessing the healthcare system, as it frequently lacks appropriate forms of communication for providing adequate information and it is not designed to address their cognitive, emotional regulation, and sensory needs (6, 9). Chiri and collaborators (10) reported that 12% of autistic individuals had at least one unmet health care need including medical care, dental care, mental health care, and prescriptions over the course of a year.

Autistic individuals may encounter difficulties in accessing healthcare, partly because their specific sensory and communication characteristics are often not fully accommodated in medical environments. Indeed, healthcare settings are frequently characterized by heightened sensory stimuli such as neon lighting, reflective surfaces, and background noise which can interfere with procedures and reduce compliance (11). Adapting healthcare environments to minimize sensory load and incorporating alternative communication strategies alongside traditional verbal methods may represent effective approaches to mitigating these barriers and ensuring the delivery of high-quality care (11).

A major barrier to healthcare access is communication, since difficulties in both receptive and expressive language can hinder the ability to seek, understand, and engage with care (12–14). Autistic individuals frequently face challenges in expressive, receptive, and pragmatic language compared to neurotypical peers (15). Communication difficulties in healthcare settings should be considered not only in relation to the communication needs of autistic individuals, but also in light of healthcare providers that may be not fully prepared to communicate both openly and through alternative modes of communication beyond oral-verbal interaction with autistic individuals (16, 17).

In healthcare contexts, communication difficulties may contribute to elevated stress and anxiety during medical procedures among autistic individuals (18). Variations in communication can make it less straightforward for autistic individuals to express their needs, especially in the context of environmental barriers, thereby contributing to emotional dysregulation (19). Indeed, many autistic children experience medical fears and phobias, such as fear of medical devices or routine exams, which are often expressed through non-compliant behaviors, intense emotional reactions, or avoidance when exposed to certain stimuli (20, 21). Of note, children, regardless of the presence of a neurodevelopmental condition, frequently experience fear and anxiety in response to medical procedures (22–24). Providing clear and age-appropriate preparatory information (i.e., using different tools tailored to the child’s age and level of comprehension, such as instructional videos and oral or written summaries) can help children regulate their emotions and cope more effectively with healthcare procedures (22).

Healthcare professionals, on the other hand, may feel ill-equipped to understand and meet the unique needs of these patients (25, 26). All of this may contribute to poorer health outcomes, increased emergency visits, and higher hospitalization rates for conditions otherwise manageable in outpatient care (25–29).

### 1.1 Communication barriers to inclusive clinical research protocols

Communication barriers often hinder healthcare professionals from effectively implementing clinical research techniques with autistic children and individuals with other neurodevelopmental conditions, due to a limited understanding of their specific needs (11, 30, 31). These challenges can exacerbate fear of equipment, reduce compliance, and limit participation in clinical trials (2). As a result, individuals with neurodevelopmental difficulties may be excluded from studies, introducing biases that perpetuate social inequities (32, 33). The underrepresentation bias in clinical trials, especially in pediatric research, is a significant issue.

Despite the potential for participation, an estimated 90% of children with neurodevelopmental conditions are excluded from research procedures, even though adequate support tools like visual aids could facilitate their inclusion (32). For example, research on ASD often focuses on autistic individuals with preserved language and cognitive abilities, excluding those with learning and behavioral difficulties due to concerns about their noncompliance (34).

Reliable research methodologies are essential for developing tailored supports that respond to the diverse needs and experiences of autistic individuals (35).

In recent decades, Non-Invasive Brain Stimulation (NIBS) techniques are attracting growing interest for the possibility to tackle at circuits’ level the pathophysiology of the different diseases (36). Among NIBS, Transcranial Direct Current Stimulation (tDCS) has emerged as a novel, cost-effective and research-based therapeutic technique for NDDs (37–41). tDCS is considered a safe and painless technique, typically associated with minor and transient effects (e.g., tingling) (42). To ensure high research standards, Brunoni and collaborators (43) developed a questionnaire to monitor tDCS safety. The tolerability and the safety of research techniques are essential for evaluating research protocols as promising for translation from research settings to clinical practice (44) tDCS involves the noninvasive application of low-amplitude direct current (0.5 to 2 mA) through at least one electrode (anode or cathode) placed over specific brain regions to modulate cortical plasticity (45), inducing subthreshold modulation of neuronal membrane excitability without generating action potentials (46).

NIBS techniques, such as tDCS, have been recently proposed in pediatric populations to explore its effect on brain plasticity as well as to treat related behavioral problems (32). Further, these techniques have also been applied to individuals with neurodevelopmental conditions, including ASD (47, 48). For example, existing studies showed improvements in language skills, working memory, autistic features severity, theory of mind, withdrawal, emotion regulation and challenging behaviors in autistic children following tDCS sessions (47, 48). Additional improvements have also been documented in stereotyped behaviors and motor skills (49, 50). Although several studies reported potential benefits of NIBS for autistic individuals, these techniques are not currently classified as an evidence-based practice by the National Clearinghouse on Autism Evidence and Practice (51).

Given the potential benefits of NIBS for ASD, it is valuable to implement these techniques (41) and to explore all the available strategies to optimize their application. This includes ensuring proper preparation, engaging caregivers actively, utilizing behavioral strategies, and fostering effective communication (52). While restraint, sedation, or anesthesia may address non-compliant behaviors in research settings, these methods risk altering test results and increasing frustration. Behavioral approaches, such as non-contingent reinforcement and differential reinforcement, may offer effective alternatives (18, 53–55). Specifically addressing communication challenges, the use of Augmentative and Alternative Communication (AAC) presents a valuable opportunity to enhance inclusive clinical and research practices and foster compliance among autistic individuals.

### 1.2 Strategies to improve communication barriers: focus on alternative and augmentative communication

AAC aims to improve communication by enhancing functional communication skills, language, social competence, and natural speech in individuals of all ages, regardless of the presence of a clinical condition (56, 57). AAC deals with two different meanings: augmentative, which applies to individuals with existing speech but limited intelligibility or expressive capabilities, and alternative to verbal language (58, 59). AAC encompasses various tools and strategies from sign language to external devices, such as alphabet boards, picture exchange communication systems (PECS), pictograms, and speech-generating devices (60).

Direct modeling in real life situations is the most effective method to provide education on AAC (58).

When teaching the use of AAC, healthcare providers have to accompany their language by touching the symbol corresponding to the spoken word or using gestures to compose messages. Depending on the chosen AAC system, the healthcare provider may only touch or exchange pictograms with the child (e.g., PECS). Additionally, caregivers should join the introduction to AAC to familiarize with these tools with the aim of implementing communication opportunities in all life contexts (58).

AAC boosts contextual language understanding, creating communicative opportunities, and reducing interactions’ misunderstandings. Consequently, AAC reduces communication barriers in various settings, such as healthcare settings (61). Moreover, AAC has the potential to increase motivation and decrease challenging behaviors (58, 62, 63).

Given the frequent medical co-occurrences associated with ASD and the communication barriers that often interfere with appropriate healthcare management, it is essential to synthesize current knowledge on the potential efficacy of AAC in promoting compliance to clinical procedures among autistic children. Of note, compliance should be conceptualized not as passive adherence, but as the outcome of accessible and person-centered communication supporting autistic individuals’ agency. This involves ensuring understanding of clinical protocols, providing tools for autistic individuals to express their needs and concerns, and supporting providers in delivering explanations aligned with autistic individuals’ needs. Furthermore, considering recent advancements in innovative and emerging interventions aimed at improving the quality of life of autistic children and their families, it is equally important to synthesize current literature on the effectiveness of AAC in facilitating the implementation of novel clinical and research procedures.

Therefore, our primary objective was to conduct a narrative literature review to synthesize the existing research on the use of AAC tools to improve compliance in general healthcare settings for autistic youth. Subsequently, we performed a second literature search to specifically synthesize the existing research on the application of AAC tools in supporting brain stimulation protocols for autistic children.

The present narrative review has addressed the following research questions:

1. Are AAC aids used in healthcare practice with benefits for autistic children’s medical compliance?
2. Have AAC aids been employed to address the compliance of autistic children during brain stimulation research procedures?

Based on the findings from the literature review, we developed a set of visual aids designed to assist the implementation of brain stimulation procedures for autistic youth. Developing AAC stimuli for such clinical research settings is essential for enhancing inclusion by reducing communication barriers and improving research feasibility.

## 2 Methods

### 2.1 Search strategy

An extensive literature search has been carried out for the current narrative review using the databases “PubMed” and “PsycInfo” without starting time restrictions, until 11 February 2026.

For the first search aim the following expressions were used: (augmentative and alternative communication OR visual schedule OR picture schedules OR pictures OR AAC OR PECS OR picture exchange communication system) AND (autis*) AND (health care OR compliance OR medical setting).

For the second search question, a different set of terms was used, specifically (augmentative and alternative communication OR visual schedule OR picture schedules OR pictures OR AAC OR PECS OR picture exchange communication system) AND (autis*) AND (neuromodulation OR brain stimulation OR tDCS).

The search terms used refers to the titles and abstracts.

After conducting the two searches and exploring the promising effectiveness of AAC in supporting the communication needs of autistic individuals in healthcare settings, we developed a set of pictograms aimed at enhancing the inclusiveness and feasibility of neuromodulation techniques for autistic individuals.

### 2.2 Study selection

The first, second, and third authors independently conducted the literature search, screened the titles and abstracts of potentially eligible studies, reviewed the full texts, and extracted descriptive data, with each author responsible for a subset of studies. The review process was conducted under non-blind conditions, with any uncertainty about whether to include or exclude a study resolved through discussion and consensus among the reviewers.

For the first research aim, articles were screened based on the eligibility criteria summarized in **Table 1**. No time restrictions were applied, allowing for the inclusion of all studies published to date on the topic.

**Table 1:** Eligibility criteria for the first research aim.

Inclusion criteria	Exclusion criteria
Studies written in English	Reviews, meta-analyses, dissertations, study protocols, case reports, book chapters, clinical practice guidelines
Studies including autistic children and adolescents	Studies solely involving non-autistic children and adolescents or autistic adults (e.g., neurotypical population, individuals with a genetic syndrome)
Studies focusing on the use of visual aids during healthcare procedures and involving autistic children and adolescents	Studies not focusing on the use of visual during healthcare procedures and involving autistic children and adolescents (e.g., articles focusing on epidemiologic aspects, conducted in the educational context)

To address the second research question, studies were included if: i) they focused on the use of NIBS in autistic children and adolescents; ii) they specifically examined the use of visual aids during NIBS procedures. No restrictions were applied regarding publication date. Only original research articles written in English were considered, while literature reviews and meta-analyses were excluded.

## 3 Results

### 3.1 Research question 1

#### 3.1.1 Study selection

**Figure 1:**
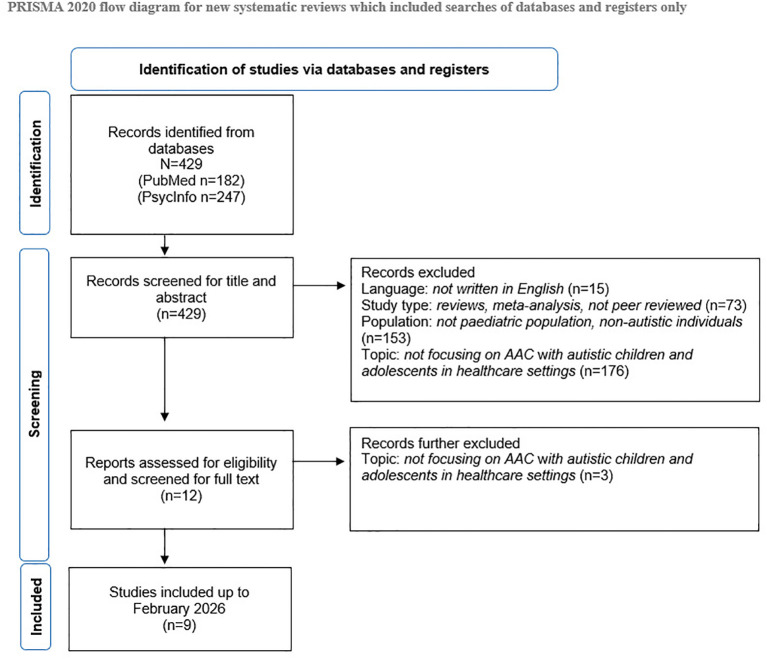
Flowchart of the selection process.

**Figure 1** details the flowchart of the selection process. The first search algorithm yielded 429 records, which were screened based on title and abstract. Of these, 417 were excluded for failing to meet the eligibility criteria (language: n = 15; population: n=153; study type: n = 73; off-topic: n = 176). The remaining 12 articles underwent full-text screening, of them 3 were further excluded (off-topic: n=3) while 9 records were subsequently included in the present narrative review.

#### 3.1.2 Summary of study characteristics

The 9 studies included in the present narrative review were conducted in 7 different countries: Brazil (n=2), Canada (n=1), France (n=1), Norway (n=1), Sweden (n=1), South Africa (n=1), and USA (n=2). Additionally, 8 adopted a cross-sectional study design while 1 study was longitudinal. Further, the majority of the studies (n=8) included in this narrative review were conducted in the dentistry context.

#### 3.1.3 Augmentative and Alternative Communication and healthcare for autistic youth

Pilebro and Bäckman (64) developed a set of visual aids to introduce the dental care context to autistic preschoolers. The study involved 32 autistic children, who were divided into two groups: 16 children used AAC visual supports in the dental context, while the remaining 16 were the control group and did not receive any visual supports. The results showed that children who were provided with visual aids demonstrated significantly greater cooperation during dental treatment compared to autistics in the control group. These findings suggest that visual aids represent a potential tool for preparing autistic children for dental care.

Isong and collaborators (65) used high tech AAC (video) to increase compliance and reduce fear during dental visits for 80 autistic youth (age range 7–17 years; 81% males and 19% females). Participants were grouped according to different conditions: video peer modeling only (i.e. video of a child undergoing a preventive dental care visit from arriving at the clinic to receiving oral treatment), video goggles only, video peer modeling plus video goggles, and control group. Results showed decreased anxiety levels, detected by the Venham Anxiety and Behavior Scale (66), along with more adaptive behaviors for video peer modeling plus video goggles condition (0.8 points). Also, children who watched the video peer more than once showed decreased anxiety levels.

Subsequently, Mah and Tsang (67) explored the use of aided AAC to explain dental procedures involving 14 autistic males (aged 4–8 years) who had been non-compliant during previous oral examinations. Half of the sample used aided AAC, while the remaining children the tell-show-do method (a teaching strategy that involves explaining, demonstrating, and allowing the learner to practice). Results showed statistically significant correlations between behavioral distress (e.g., screaming, crying, and escaping), assessed with the Child-Adult Medical Procedure Interaction Scale-Short Form (68), and the number of completed steps and the time required to end each dental procedure. Furthermore, lower distress levels were associated with a greater number of successful dental examinations. While the number of steps completed increased over repeated weekly appointments in both groups, children who received visual cues completed oral medication administration in a shorter amount of time.

Zink and colleagues (69) investigated potential improvements using tailored pictograms during oral checkups in 26 autistic children (mean age 10 ± 3.3 years; 22 males and 4 females). Of them, 13 children had no prior experience with dental treatments (G1), while 12 had previous experience (G2). Results showed that G1 required significantly less time to perform specific medical steps using PECS compared to G2.

Another study conducted by Zink and collaborators (70) compared high tech AAC (e.g., app with images linked to audio descriptions) and low tech AAC (PECS) in enhancing communication during dental examinations involving 40 autistic youth (aged 9 to 15 years; 38 males and 2 females), equally divided into 2 groups. Results indicated statistically significant differences in the number of attempts and the number of appointments for dental exams, with the app condition showing more improvements. Authors also suggested that a decrease in the number of appointments could potentially reduce reliance on general anesthesia for outpatient procedures.

Lefer and colleagues (71) explored that effectiveness of aided AAC containing 6 dental pictograms with a psychoeducational program on oral health involving 52 autistic children (mean age 10.2 years; 45 males and 7 females). Children underwent 5 dental care examinations (T0, T1, T2, T3, and T4) over 8-months in which their compliance with dental steps and their anxiety levels by using the Frankl Behavior Rating Scale (72) were monitored. Results showed improvements in tolerance toward dental examination: the estimated percentage ranging from 2 to 17% of children who did not perform the first four steps at T0 decreased to 0% by the end of the search. The percentage of children achieving the initial 4 steps increased over time from a range of 37-79% (T0) to 75-86.5% (T4). Further, the percentage of individuals mastering the entire exam procedure increased from 25% at T0 to 65.4% at T4. Additionally, the lack of perceived anxiety increased to 36.5% at T1, 51.9% at T2 and T3, and 59.6% at T4. The Global scores for compliance and anxiety levels showed significant differences between T0 and T1, as well as between T0 and T4. However, the results were not controlled for a familiarity effect due to the repeated exposure and practice in the setting.

Naidoo and colleagues (73) investigated which AAC stimuli are most effective for creating communication boards to describe dental examinations for autistic children aged 7–14. Results showed that, after selecting the most representative and child-friendly dental, a survey of dental professionals revealed that 80% considered AAC useful for explaining treatment plans to autistic children, and 60% believed it helped improve children’s compliance.

Myhren and collaborators (74) explored the efficacy of aided AAC in improving oral checkup and reducing the use of general anesthesia or sedation in 17 autistic children (aged 6–13 years; 13 males and 4 females). Of autistic children and adolescents involved, 12 had partially or fully adequate language development while 15 showed several sensory differences (e.g., sound, smell, and taste). Participants underwent a “habituation program” for gradually introducing children to various dental examinations (e.g., X-ray). Results showed that the 82% of autistic children completed all dental examination’s steps and the 59% underwent X-ray with good compliance, evaluated by the Frankl Behavior Rating Scale (72). Of note, the presence or not of the same dental provider did not influence autistic children’s cooperation. At one-year follow-up the compliance was preserved, except for one child.

Only one study conducted by Chebuhar and collaborators (75) focused on the use of AAC for sustaining general medical examinations. Authors examined the implementation of aided AAC sets with 17 autistic children during different healthcare examinations (e.g., administration of oxygen, x-ray, EEG, injections, finger sticks). Results underscored that almost 87.5% of the medical staff and 77.8% of caregivers interviewed reported a decrease in children’s anxiety levels and more tolerable visits in terms of exhibiting challenging behaviors when AAC boards were used. Further, parents expressed strong support for the use of visual communication boards, noting that these tools provided a sense of reassurance and contributed to a more positive clinical experience.

### 3.2 Research question 2

#### 3.2.1 Study selection

The second algorithm yielded 210 results. All retrieved articles were screened based on their titles and abstracts. However, none of the articles focused on AAC, compliance, or research procedures, nor on NIBS in autistic children and adolescents.

## 4 Augmentative and Alternative Communication protocol development for brain stimulation

To the best of our knowledge, no AAC protocol has yet been developed to support brain stimulation procedures. Therefore, the creation of AAC aids represents a unique opportunity to better explain research stages to children with NDDs. Although brain stimulation is an evidence-based research practice increasingly known among clinicians and researchers, it is possible that brain stimulation procedures and tools will not be familiar to children with NNDs and their families. Thereby, specific support, such as visual aids, may be valuable for effectively implementing clinical-research procedures.

### 4.1 Augmentative and Alternative Communication aids

Pictures depicting different clinical and research procedures have been specifically developed by a team made up of four psychologists and a speech pathologist and assembled into visual schedules.

**Figure 2:**
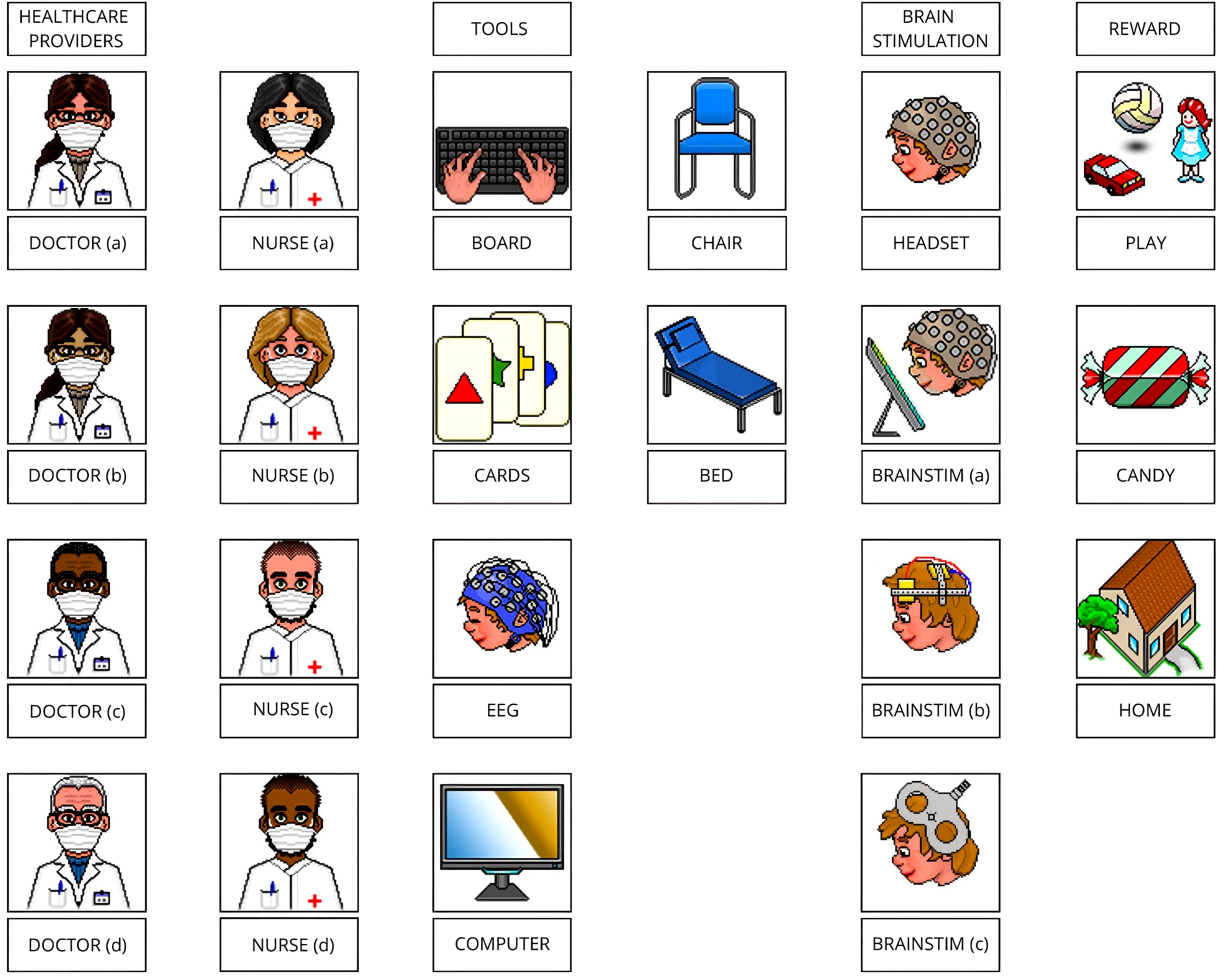
Set of AAC pictograms for illustrating brain stimulation procedures. Brainstim **(A)**, High-Definition Transcranical Direct current Stimulation; Brainstim **(B)**, Transcranical Direct current Stimulation; Brainstim **(C)**, Transcranial Magnetic Stimulation.

Features of realism, iconicity (association between a symbol and its referent), ambiguity, complexity, figure-ground differential, perceptual distinctness, acceptability, efficiency, color and size discriminate pictograms were followed (57, 76, 77). Specific attention was placed on guessability or transparency to conversational partners and ease of acquisition by combining pictures with traditional orthography (e.g., printed words) (57). Visual aids were specifically created by a graphic designer who illustrated colored pictograms based on the instructions provided by a speech pathologist (**Figure 2**). Two psychologists and a speech pathologist selected the words that best describe the images.

**Figure 3:**
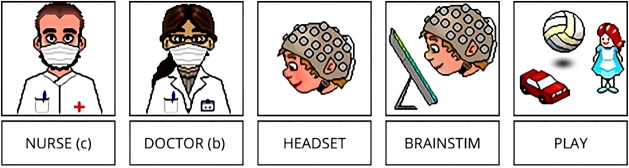
AAC sequence depicting a brain stimulation session, from the moment of acceptance to putting on the headset, and ending with the final reward.

Pictograms (3x3 cm) have been arranged in a horizontal format with words printed below them. To depict day hospital activities, specifically involving brain stimulation, a sequence of four to six pictures has been attached from left to right. Each picture has Velcro on its back, facilitating the creation of sequences (**Figure 3**).

Since neuromodulation techniques are often integrated with electroencephalography (EEG) to evaluate their outcomes (78), the developed AAC protocol includes pictograms of neuromodulation techniques as well as one representing EEG procedure.

**Figure 4:**
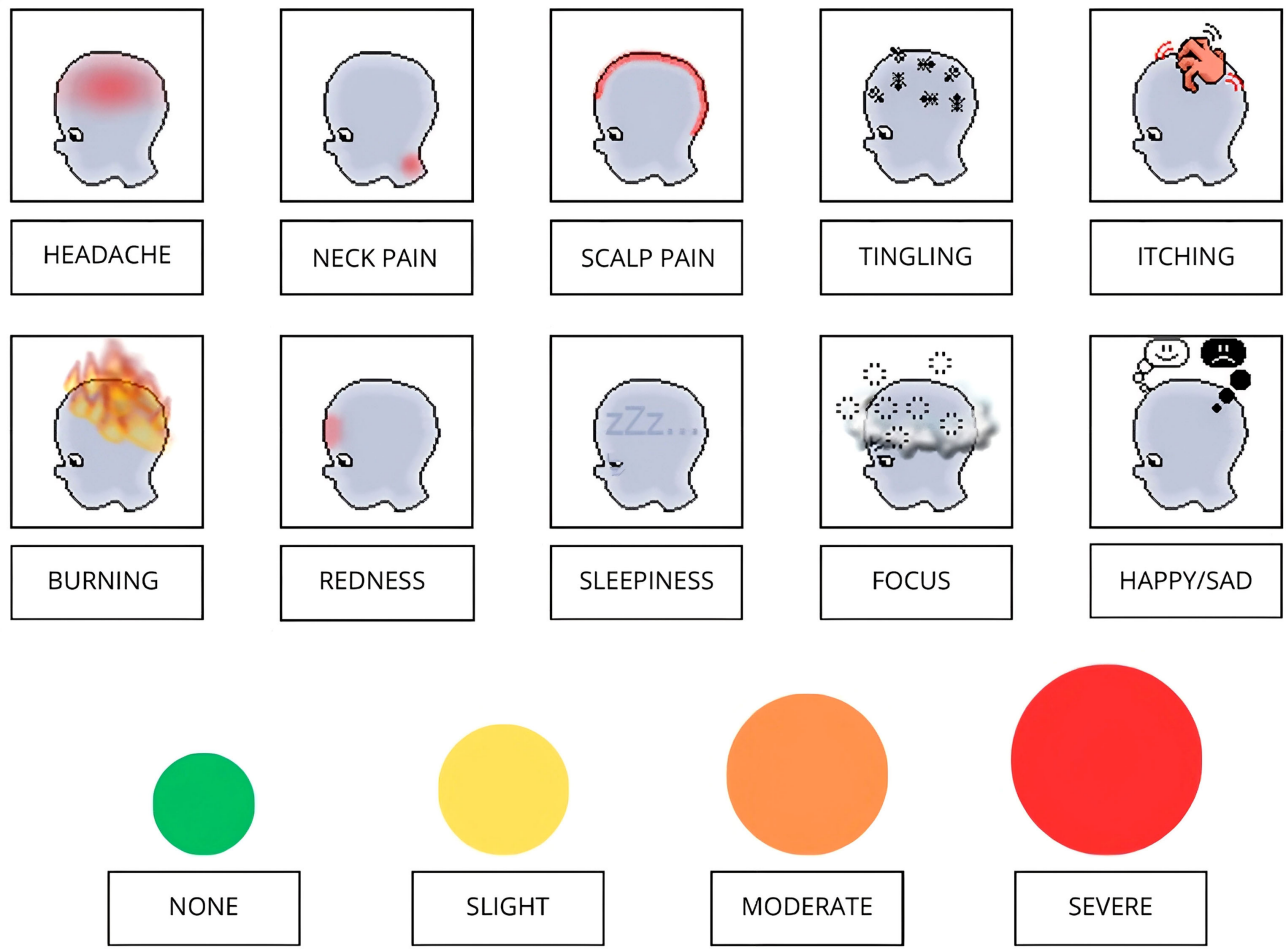
Visual aids illustrating potential stimulation effects and a chart indicating sensation intensity.

Furthermore, to ensure an inclusive research practice, we created additional visual aids to monitor potential sensations associated with brain stimulation (e.g., headache, itching, neck pain). Specifically, pictograms were developed to translate the Safety Questionnaire (43) into AAC, facilitating the collection of reliable information from children with NDDs after undergoing brain stimulation. Visual aids depicting possible effects of brain stimulation along with a chart to express the gradient of sensations were developed (**Figure 4**).

## 5 Discussion

The first aim of this narrative review was to synthesize the current literature on the potential of AAC in fostering compliance with medical procedures in autistic children and adolescents. Most of the included studies (N = 8) focused on dental care, aiming to encourage pediatric dentists to use AAC to alleviate distress, anxiety, and sustain compliance. The strong emphasis on the use of AAC in dental settings may be attributed to several contextual factors. Dental care typically requires patients to attend multiple appointments, which, although brief, occur periodically and often involve procedures that can be painful or uncomfortable. Additionally, dental equipment tends to generate sensory input. Consequently, AAC represents an effective tool for explaining procedures, particularly with regard to sequencing and timing. This aligns with the findings of Niro and colleagues (73), who showed that autistic features such as repetitive behaviors associated with the need for routines as well as sensory sensitivities can make healthcare environments challenging for autistic children. As a result, exposure to intense sensory stimuli, unexpected disruptions to established routines, and waiting times may contribute to non-compliant behaviors (79).

The reviewed studies also highlight the crucial role of aided AAC in improving tolerance during healthcare procedures, enhancing the understanding of medical procedures, and reducing anxiety, distress, and challenging behaviors, thus promoting medical compliance (71, 75). These findings align with the study of McTee and collaborators (80) in which they discuss the potential of visual aids for fostering autistic children’s compliance during audiological exams and reducing procedure-related anxiety.

Aided AAC allows for clear, simple explanations of procedures, helping children and parents prepare and anticipate medical steps (65, 69, 71). AAC tools also support parents by providing reassurance and assisting in managing challenging behaviors, making the healthcare experience less stressful (75).

The second aim of the current work was to conduct a literature review specifically focused on the use of AAC to support brain stimulation procedures. Our literature review failed to identify studies focused on the use of AAC to foster compliance with clinical-research procedures involving brain stimulation in autistic children and adolescents. Scientific research aims to improve the well-being of all individuals by advancing knowledge and leveraging new technologies to transform healthcare and address neurodivergent development (81).

Although NIBS are relatively feasible to apply, they require specific equipment that children are often unfamiliar with and that stimulates distinct sensory patterns, such as the tactile one. Furthermore, the application of NIBS requires patient compliance to follow procedural steps (e.g., entering the room, sitting still, wearing a headset, performing tasks) and for a moderately long duration. For instance, tDCS protocols for ASD typically last at least 20 minutes (82).

Interestingly, our review found that AAC is an effective tool to enhance compliance in oral care settings. Although the two contexts (i.e., dental care vs NIBS) differ in terms of objectives, they share the features that the equipment is unfamiliar, both stimulate sensory experiences, and require patients to complete multiple procedural steps. Basing on these similarities, it is supposable that the use of AAC could extend to NIBS procedures to facilitate their implementation in research settings.

Researchers should balance scientific rigor with an inclusive, individualized approach that considers the uniqueness of each participant, ensuring both large-scale replication and a human-centered perspective to advance progress while managing feasibility and time constraints. Recent advancements in research in the application of NIBS within autistic individuals highlight the importance of considering their unique needs during the research process. Further, the acceptability of new procedures should be explored from the perspectives of the autistic community.

Several limitations of the included studies should be noted: none reported randomization processes or sample size calculations, and most had small sample sizes. Additionally, different types of AAC aids were used for similar procedures, limiting replicability. Most studies focused on qualitative outcomes, whereas quantitative methods would offer more valuable insights. These issues affect the generalizability of the results. Further studies are needed to refine AAC’s application in healthcare and research settings (83).

Notably, none of the studies explicitly focused on pain assessment. Given the challenges of obtaining verbal information from autistic children and adolescents, more attention should be given to customizing pain-related vocabulary and pictograms (84).

To sum, our literature review highlights that AAC can help overcome communication barriers for autistic children, fostering a positive environment that benefits both children and healthcare professionals by supporting interactions and facilitating efficient intervention procedures.

These advantages can also be extended to brain stimulation procedures: applying aided AAC within brain stimulation procedures offers a promising approach for achieving an inclusive and patient-centered research methodology. This practice would enable autistic children and adolescents, or more broadly individuals with neurodevelopmental difficulties, to more effectively express themselves and comprehend their experiences by accommodating the full spectrum of their verbal and communicative abilities.

The AAC aids we developed were specifically designed to promote more inclusive clinical research procedures. Possible applications of these aids may include both children who are already familiar with AAC systems and those who are not yet experienced users but may benefit from their introduction. For participants without prior AAC experience, a preliminary familiarization and training phase should be conducted to ensure they can effectively use the communication supports during the brain stimulation sessions. One of the two stimulus sets include pictograms representing various professional roles, activities, and tools, which help illustrate some key aspects of the assessment procedures in clinical research protocols. This approach aims to mitigate the aforementioned underrepresentation bias by encouraging effective participation and compliance from children with NDDs, regardless of their language or learning abilities. Since participants are required to provide verbal feedback regarding the sensations associated with brain stimulation at the end of each tDCS session, a second set of stimuli was created to visually represent each potential sensation linked to brain stimulation and included in the Safety Questionnaire (43). The use of visual aids to monitor potential adverse effects of tDCS enables the direct collection of insights from children who may struggle with communication, overcoming the common tendency to infer information about the well-being, sensations, and feelings of individuals with communication difficulties through their caregivers (25). The application of our visual aids may be recommended for individuals with various clinical conditions, across all age groups and language proficiencies, as outlined in the AAC manual (56, 57).

Nonetheless, several limitations should be acknowledged when considering the proposed AAC protocol for autistic youth in brain stimulation settings. First, the development of the AAC aids did not involve consultation with the autistic community in the selection of visual stimuli. Second, there is a lack of assessment of the comprehension and use of pictograms among autistic children and adolescents. Finally, no pilot or feasibility studies were conducted to evaluate the usability of the developed AAC tools.

Finally, the use of our AAC aids responds to the need for greater equity, helping reduce disparities in attending interventions and supportive services for autistic individuals and their families within both clinical practice and research. Furthermore, the possibility of implementing AAC aids according to different identities and languages makes them valuable cross-cultural tools that can be used effectively to reduce disparities (85, 86).

## 6 Conclusion

Although the use of AAC to sustain brain stimulation procedures has not yet been explored, the present study underscores the advantages of using aided AAC to improve compliance in autistic children and adolescents during medical examinations. This review lays the groundwork for developing future AAC protocols to enhance healthcare quality and equity in clinical and research settings. In future research, the development of our AAC protocol should include implementation through consultation with the autistic community regarding the visual stimuli used, followed by a pilot and feasibility studies to assess usability. Specifically, these studies should evaluate the comprehension and effective use of pictograms in autistic children and adolescents, ensuring that they are both accessible and functionally appropriate for the target population. If pictograms are found to be usable and effective for this population, these visual supports could be extended to families, who may use them to create social stories to better prepare their children in advance for upcoming situations. Moreover, the proposed AAC aids would contribute to filling the gap on using visual aids among researchers and clinicians.

Ensuring compliance with research procedures is essential for delivering high-quality medical care, as it is grounded in research itself. Indeed, clinical trials represent the most robust studies for advancing scientific knowledge regarding the effectiveness of new drugs or interventions.

Adopting a restrictive research approach can have significant ethical, social, and scientific implications (32), making the implementation of AAC protocols crucial for respecting ethical principles while advancing scientific understanding.
